# Supplementary material for: Sonographic Features Differentiating Follicular Thyroid Cancer from Follicular Adenoma–A Meta-Analysis
Source: Cancers (Basel). 2021 Feb 24;13(5):938. doi: 10.3390/cancers13050938 (PMC7956257; doi:10.3390/cancers13050938)
Supplement: Supplementary file 1 [file cancers-13-00938-s001.pdf]

Supplementary materials: Sonographic Features Differentiating Follicular Thyroid Cancer from Follicular Adenoma–A Meta-Analysis

Martyna Borowczyk, Kosma Woliński, Barbara Więckowska, Elżbieta Jodłowska-Siewert, Ewelina Szczepanek-Parulska, Frederik A. Verburg and Marek Ruchała

Table S1a. The results of odds rations (OR) of each study in increasing the risk of nodule malignancy for tumor protrusion with 95% confidence intervals and forest plots.

Author	Year	n	OR [95%CI]	Weight
Sillery JC et al.	2010	102	3.18 [0.13, 79.96]	17.8%
Kobayashi K et al.	2016	531	13.11 [2.93, 58.77]	82.2%
**Overall OR**			**10.19 [2.62, 39.71]**	

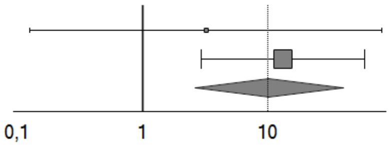

Odds Ratio (log –scale)

Test of heterogeneity
I^2=0.0%, p=0.4350

Egger's asymmetry test
b Egger=NA, p=NA

NA- not applicable (too few data)

Table S1b. The number of patients with true positive (TP), false negative (FN), false positive (FP), and true negative (TN) results presenting the estimates of sensitivity, specificity, positive predictive value (PPV), and negative predictive value (NPV with 95% confidence intervals of each study for tumor protrusion.

Author	Year	n	TP	FN	FP	TN	Sensitivity [95%CI]	Specificity [95%CI]	PPV [95%CI]	NPV [95%CI]
Sillery JC et al.	2010	102	1	49	0	52	0.02 [0.00, 0.11]	1.00 [0.93, 1.00]	1.00 [0.02, 1.00]	0.51 [0.41, 0.62]
Kobayashi K et al.	2016	531	13	171	2	345	0.07 [0.04, 0.12]	0.99 [0.98, 1.00]	0.87 [0.6, 0.98]	0.67 [0.63, 0.71]
**Overall sensitivity, specificity, PPV, NPV**							**0.06 [0.03, 0.09]**	**1.00 [0.99, 1.00]**	**0.96 [0.7, 1.00]**	**0.64 [0.61, 0.68]**

HSROC overall sensitivity and specificity are missing because HSROC requires a minimum of four studies

Table S2a. The results of odds rations (OR) of each study in increasing the risk of nodule malignancy when microcalcifications or mixed type (coexisting micro- and macrocalcifications) are present in the ultrasound with 95% confidence intervals and forest plots.

Author	Year	n	OR [95%CI]	Weight
Seo HS et al.	2009	126	5.8 [1.23, 27.36]	16.82%
Lai X et al.	2013	111	8.4 [2.48, 28.46]	27.18%
Pompili G et al.	2013	102	3.31 [0.28, 39.12]	6.63%
Aydın Y et al.	2014	88	0.43 [0.02, 8.20]	4.69%
Yoon JH et al.	2014	177	20.59 [2.05, 206.81]	7.60%
Jeong SH et al.	2016	178	0.97 [0.05, 19.5]	4.51%
Kuru B et al.	2018	139	9.69 [2.61, 36.01]	23.50%
Kuo TC et al.	2019	188	2.88 [0.18, 46.86]	5.19%
Liu BJ et al.	2020	90	6.82 [0.27, 172.69]	3.87%
	**Overall OR**		**6.09 [3.22, 11.5]**	

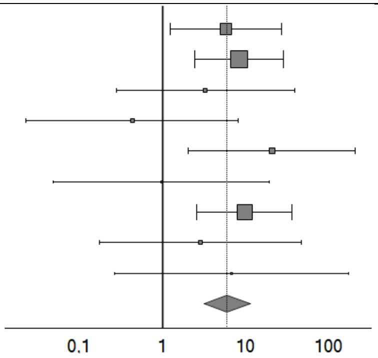

Test of heterogeneity
$I^2 = 0\%$, p = 0.5494

Egger's asymmetry test
b Egger = -1.50, p = 0.0800

Table S2b. The number of patients with true positive (TP), false negative (FN), false positive (FP), and true negative (TN) results presenting the estimates of sensitivity, specificity, positive predictive value (PPV), and negative predictive value (NPV with 95% confidence intervals of each study for microcalcifications or mixed type (coexisting micro- and macrocalcifications) in the ultrasound.

Author	Year	n	TP	FN	FP	TN	Sensitivity [95%CI]	Specificity [95%CI]	PPV [95%CI]	NPV [95%CI]
Seo HS et al.	2009	126	11	55	2	58	0.17 [0.09, 0.28]	0.97 [0.88, 1.00]	0.85 [0.55, 0.98]	0.51 [0.42, 0.61]
Lai X et al.	2013	111	12	25	4	70	0.32 [0.18, 0.50]	0.95 [0.87, 0.99]	0.75 [0.48, 0.93]	0.74 [0.64, 0.82]
Pompili G et al.	2013	102	1	13	2	86	0.07 [0.00, 0.34]	0.98 [0.92, 1.00]	0.33 [0.01, 0.91]	0.87 [0.79, 0.93]
Aydın Y et al.	2014	88	0	6	12	70	0.00 [0.00, 0.46]	0.85 [0.76, 0.92]	0.00 [0.00, 0.26]	0.92 [0.84, 0.97]
Yoon JH et al.	2014	177	3	22	1	151	0.12 [0.03, 0.31]	0.99 [0.96, 1.00]	0.75 [0.19, 0.99]	0.87 [0.81, 0.92]
Jeong SH et al.	2016	178	0	22	3	153	0.00 [0.00, 0.15]	0.98 [0.94, 1.00]	0.00 [0.00, 0.71]	0.87 [0.82, 0.92]
Kuru B et al.	2018	139	13	38	3	85	0.25 [0.14, 0.40]	0.97 [0.90, 0.99]	0.81 [0.54, 0.96]	0.69 [0.60, 0.77]
Kuo TC et al.	2019	188	1	48	1	138	0.02 [0.00, 0.11]	0.99 [0.96, 1.00]	0.50 [0.01, 0.99]	0.74 [0.67, 0.80]
Liu BJ et al.	2020	90	1	27	0	62	0.04 [0.00, 0.18]	1.00 [0.94, 1.00]	1.00 [0.02, 1.00]	0.70 [0.59, 0.79]
**Overall sensitivity, specificity, PPV, NPV**							**0.10 [0.03, 0.19]**	**0.97 [0.95, 0.99]**	**0.53 [0.19, 0.86]**	**0.78 [0.69, 0.85]**

HSROC overall sensitivity [95%CI] = 0.1 [0.04, 0.21], HSROC overall specificity [95%CI] = 0.98 [0.95, 0.99]

Table S3a. The results of odds rations (OR) of each study in increasing the risk of nodule malignancy for irregular margins with 95% confidence intervals and forest plots.

Author	Year	n	OR [95%CI]	Weight
Seo HS et al.*	2009	126	0.81 [0.37, 1.77]	9.28%
Sillery JC et al.*	2010	102	0.57 [0.21, 1.53]	8.76%
Lee EK et al.	2012	110	3.2 [1.05, 9.73]	8.40%
Lai X et al.	2013	111	20.14 [2.41, 168.26]	5.62%
Lee KH et al.	2013	75	0.39 [0.02, 7.44]	3.95%
Lee SH et al.*	2013	66	0.27 [0.03, 2.27]	5.58%
Pompili G et al.	2013	102	15.75 [3.66, 67.7]	7.40%
Cordes M et al.	2014	57	8 [2.41, 26.57]	8.15%
Yoon JH et al.	2014	177	3.97 [1.21, 13.05]	8.18%
Cordes M et al.*	2016	200	152.06 [9.19, 2516.81]	4.19%
Jeong SH et al.	2016	178	1.6 [0.49, 5.24]	8.19%
Kuru B et al.	2018	139	3.34 [0.93, 12.03]	7.91%
Kuo TC et al.	2019	188	7.78 [1.46, 41.54]	6.79%
Liu BJ et al.	2020	90	12.73 [3.18, 50.88]	7.61%
	**Overall OR**		**3.49 [1.66, 7.35]**	

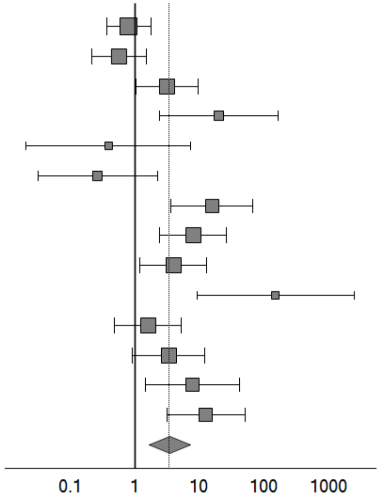

Test of heterogeneity
I^2=75.3%, p<0.0001

Egger's asymmetry test
b Egger=2.70, p=0.0980

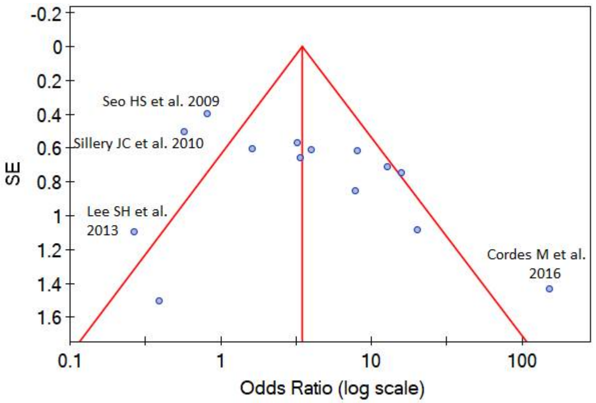

* Outleier

After exclusion of outliers: **overall OR [95%CI] = 5.11 [2.9, 8.99]**, test of heterogeneity I^2=36.59%, p=0.1156

Table S3b. The number of patients with true positive (TP), false negative (FN), false positive (FP), and true negative (TN) results presenting the estimates of sensitivity, specificity, positive predictive value (PPV), and negative predictive value (NPV with 95% confidence intervals of each study for irregular margins.

Author	Year	n	TP	FN	FP	TN	Sensitivity [95%CI]	Specificity [95%CI]	PPV [95%CI]	NPV [95%CI]
Seo HS et al. *	2009	126	17	49	18	42	0.26 [0.16, 0.38]	0.70 [0.57, 0.81]	0.49 [0.31, 0.66]	0.46 [0.36, 0.57]
Sillery JC et al. *	2010	102	8	42	13	39	0.16 [0.07, 0.29]	0.75 [0.61, 0.86]	0.38 [0.18, 0.62]	0.48 [0.37, 0.60]
Lee EK et al.	2012	110	8	25	7	70	0.24 [0.11, 0.42]	0.91 [0.82, 0.96]	0.53 [0.27, 0.79]	0.74 [0.64, 0.82]
Lai X et al.	2013	111	8	29	1	73	0.22 [0.10, 0.38]	0.99 [0.93, 1.00]	0.89 [0.52, 1.00]	0.72 [0.62, 0.80]
Lee KH et al.	2013	75	0	11	6	58	0.00 [0.00, 0.28]	0.91 [0.81, 0.96]	0.00 [0.00, 0.46]	0.84 [0.73, 0.92]
Lee SH et al. *	2013	66	1	15	10	40	0.06 [0.00, 0.30]	0.80 [0.66, 0.90]	0.09 [0.00, 0.41]	0.73 [0.59, 0.84]
Pompili G et al.	2013	102	6	8	4	84	0.43 [0.18, 0.71]	0.95 [0.89, 0.99]	0.60 [0.26, 0.88]	0.91 [0.84, 0.96]
Cordes M et al.	2014	57	18	6	9	24	0.75 [0.53, 0.90]	0.73 [0.54, 0.87]	0.67 [0.46, 0.83]	0.80 [0.61, 0.92]
Yoon JH et al.	2014	177	5	20	9	143	0.20 [0.07, 0.41]	0.94 [0.89, 0.97]	0.36 [0.13, 0.65]	0.88 [0.82, 0.92]
Cordes M et al. *	2016	200	100	0	57	43	1.00 [0.96, 1.00]	0.43 [0.33, 0.53]	0.64 [0.56, 0.71]	1.00 [0.92, 1.00]
Jeong SH et al.	2016	178	4	18	19	137	0.18 [0.05, 0.40]	0.88 [0.82, 0.93]	0.17 [0.05, 0.39]	0.88 [0.82, 0.93]
Kuru B et al.	2018	139	7	44	4	84	0.14 [0.06, 0.26]	0.95 [0.89, 0.99]	0.64 [0.31, 0.89]	0.66 [0.57, 0.74]
Kuo TC et al.	2019	188	5	44	2	137	0.10 [0.03, 0.22]	0.99 [0.95, 1.00]	0.71 [0.29, 0.96]	0.76 [0.69, 0.82]
Liu BJ et al.	2020	90	11	17	3	59	0.39 [0.22, 0.59]	0.95 [0.87, 0.99]	0.79 [0.49, 0.95]	0.78 [0.67, 0.86]
After outliers exclusion **overall sensitivity, specificity, PPV, NPV**							**0.24 [0.13, 0.37]**	**0.94 [0.90, 0.96]**	**0.53 [0.34, 0.71]**	**0.80 [0.74, 0.86]**

* Outlier

HSROC overall sensitivity [95%CI] = 0.24 [0.15, 0.37], HSROC overall specificity[95%CI] = 0.94 [0.89, 0.96] after exclusion of outliers

Table S4a. The results of odds rations (OR) of each study in increasing the risk of nodule malignancy for hypoechogenicity markedly hypoechogenic nodules with 95% confidence intervals and forest plots.

Author	Year	n	OR [95%CI]	Weight
Seo HS et al.	2009	93	1.51 [0.64, 3.57]	7.41%
Sillery JC et al.	2010	102	4.56 [1.85, 11.24]	7.22%
Lee EK et al.	2012	110	5.75 [2.36, 14.03]	7.27%
Lai X et al.	2013	111	2.86 [1.26, 6.51]	7.57%
Lee KH et al.	2013	75	6.58 [1.31, 32.95]	4.59%
Lee SH et al.*	2013	66	0.26 [0.08, 0.85]	5.99%
Pompili G et al.	2013	102	11.6 [2.44, 55.23]	4.74%
Aydın Y et al.	2014	88	5.31 [0.96, 29.24]	4.31%
Cordes M et al.	2014	57	7.59 [2.32, 24.87]	6.06%
Yoon JH et al.	2014	177	2.88 [1.20, 6.94]	7.32%
Zhang JZ et al.	2014	88	12.42 [4.46, 34.58]	6.72%
Cordes M et al..	2016	200	39.25 [2.32, 663.97]	2.18%
Jeong SH et al.	2016	178	7.42 [2.59, 21.28]	6.60%
Kuru B et al.	2018	139	4.73 [2.22, 10.09]	7.84%
Kuo TC et al.*	2019	188	1.12 [0.47, 2.69]	7.35%
Liu BJ et al.	2020	90	2.17 [0.8, 5.85]	6.84%
		**Overall OR**	**3.69 [2.3, 5.92]**	

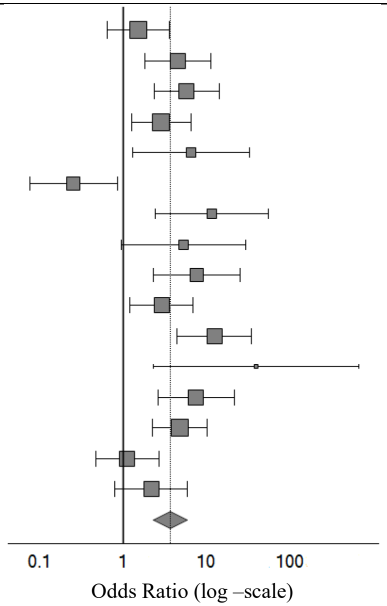

Test of heterogeneity
$I^2 = 68.1\%$, p<0.0001

Egger's asymmetry test
b Egger = 1.83, p = 0.2811

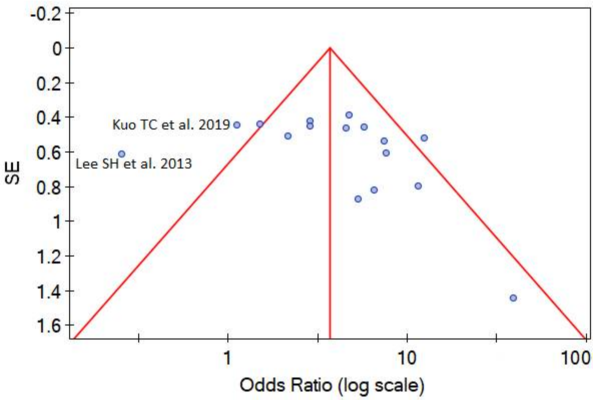

*Outlier
After exclusion of outliers: **overall OR [95%CI] = 4.59 [3.23, 6.54],** test of heterogeneity I^2 =34.96%, p = 0.0955

Table S4b. The number of patients with true positive (TP), false negative (FN), false positive (FP), and true negative (TN) results presenting the estimates of sensitivity, specificity, positive predictive value (PPV), and negative predictive value (NPV with 95% confidence intervals of each study for hypoechogenicity or markedly hypoechogenic nodules.

Author	Year	n	TP	FN	FP	TN	Sensitivity [95%CI]	Specificity [95%CI]	PPV [95%CI]	NPV [95%CI]
Seo HS et al.	2009	93	16	17	23	37	0.48 [0.31, 0.66]	0.62 [0.48, 0.74]	0.41 [0.26, 0.58]	0.69 [0.54, 0.80]
Sillery JC et al.	2010	102	41	9	26	26	0.82 [0.69, 0.91]	0.50 [0.36, 0.64]	0.61 [0.49, 0.73]	0.74 [0.57, 0.88]
Lee EK et al.	2012	110	23	10	22	55	0.70 [0.51, 0.84]	0.71 [0.60, 0.81]	0.51 [0.36, 0.66]	0.85 [0.74, 0.92]
Lai X et al.	2013	111	24	13	29	45	0.65 [0.47, 0.80]	0.61 [0.49, 0.72]	0.45 [0.32, 0.60]	0.78 [0.65, 0.87]
Lee KH et al.	2013	75	9	2	26	38	0.82 [0.48, 0.98]	0.59 [0.46, 0.71]	0.26 [0.12, 0.43]	0.95 [0.83, 0.99]
Lee SH et al.*	2013	66	5	11	32	18	0.31 [0.11, 0.59]	0.36 [0.23, 0.51]	0.14 [0.05, 0.29]	0.62 [0.42, 0.79]
Pompili G et al.	2013	102	12	2	30	58	0.86 [0.57, 0.98]	0.66 [0.55, 0.76]	0.29 [0.16, 0.45]	0.97 [0.88, 1.00]
Aydın Y et al.	2014	88	3	3	13	69	0.50 [0.12, 0.88]	0.84 [0.74, 0.91]	0.19 [0.04, 0.46]	0.96 [0.88, 0.99]
Cordes M et al.	2014	57	17	7	8	25	0.71 [0.49, 0.87]	0.76 [0.58, 0.89]	0.68 [0.46, 0.85]	0.78 [0.60, 0.91]
Yoon JH et al.	2014	177	16	9	58	94	0.64 [0.43, 0.82]	0.62 [0.54, 0.70]	0.22 [0.13, 0.33]	0.91 [0.84, 0.96]
Zhang JZ et al.	2014	88	26	10	9	43	0.72 [0.55, 0.86]	0.83 [0.70, 0.92]	0.74 [0.57, 0.88]	0.81 [0.68, 0.91]
Cordes M et al.	2016	200	100	0	84	16	1.00 [0.96, 1.00]	0.16 [0.09, 0.25]	0.54 [0.47, 0.62]	1.00 [0.79, 1.00]
Jeong SH et al.	2016	178	17	5	49	107	0.77 [0.55, 0.92]	0.69 [0.61, 0.76]	0.26 [0.16, 0.38]	0.96 [0.90, 0.99]
Kuru B et al.	2018	139	28	23	18	70	0.55 [0.40, 0.69]	0.80 [0.70, 0.87]	0.61 [0.45, 0.75]	0.75 [0.65, 0.84]
Kuo TC et al.*	2019	188	41	8	114	25	0.84 [0.7, 0.93]	0.18 [0.12, 0.25]	0.26 [0.2, 0.34]	0.76 [0.58, 0.89]
Liu BJ et al.	2020	90	21	7	36	26	0.75 [0.55, 0.89]	0.42 [0.3, 0.55]	0.37 [0.24, 0.51]	0.79 [0.61, 0.91]
**Overall sensitivity, specificity, PPV, NPV (after outliers' exclusion)**							**0.74 [0.6, 0.86]**	**0.63 [0.53, 0.73]**	**0.44 [0.35, 0.53]**	**0.87 [0.81, 0.92]**

*Outlier

HSROC overall sensitivity[95%CI] = 0.74 [0.62, 0.84], HSROC overall specificity[95%CI] = 0.63 [0.53, 0.73] after exclusion of outliers

Table S5a. The results of odds rations (OR) of each study in increasing the risk of nodule malignancy for irregular shape with 95% confidence intervals and forest plots.

Author	Year	n	OR [95%CI]	Weight
Lai X et al.	2013	111	8.40 [2.48, 28.46]	22.11%
Cordes M et al.	2014	57	0.66 [0.11, 3.93]	18.34%
Cordes M et al.*	2016	200	32.25 [14.82, 70.19]	24.76%
Jeong SH et al.	2016	178	3.67 [0.32, 42.21]	14.34%
Kuo TC et al.	2019	188	5.15 [1.18, 22.43]	20.44%
Overall OR			**5.9 [1.53, 22.82]**	

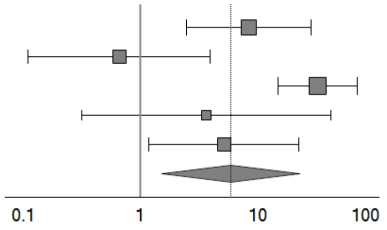

Test of heterogeneity
I^2=78.8%, p=0.0008

Egger's asymmetry test
b Egger=-4.53, p=0.0589

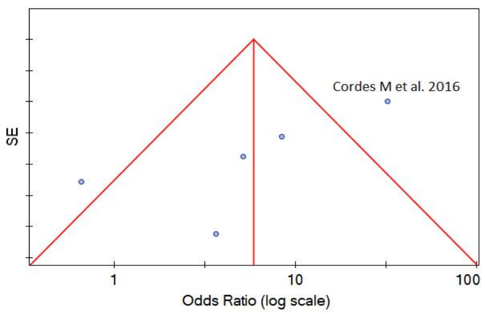

* Outlier

After exclusion of outliers: **overall OR [95%CI] = 3.6 [1.19, 10.92]**, test of heterogeneity I^2=45.0%, p=0.1415

Table S5b. The number of patients with true positive (TP), false negative (FN), false positive (FP), and true negative (TN) results presenting the estimates of sensitivity, specificity, positive predictive value (PPV), and negative predictive value (NPV with 95% confidence intervals of each study for irregular shape.

Author	Year	n	TP	FN	FP	TN	Sensitivity [95%CI]	Specificity [95%CI]	PPV [95%CI]	NPV [95%CI]
Lai X et al.	2013	111	12	25	4	70	0.32 [0.18, 0.50]	0.95 [0.87, 0.99]	0.75 [0.48, 0.93]	0.74 [0.64, 0.82]
Cordes M et al.	2014	57	2	22	4	29	0.08 [0.01, 0.27]	0.88 [0.72, 0.97]	0.33 [0.04, 0.78]	0.57 [0.42, 0.71]
Cordes M et al. *	2016	200	86	14	16	84	0.86 [0.78, 0.92]	0.84 [0.75, 0.91]	0.84 [0.76, 0.91]	0.86 [0.77, 0.92]
Jeong SH et al.	2016	178	1	21	2	154	0.05 [0.00, 0.23]	0.99 [0.95, 1.00]	0.33 [0.01, 0.91]	0.88 [0.82, 0.92]
Kuo TC et al.	2019	188	5	44	3	136	0.10 [0.03, 0.22]	0.98 [0.94, 1.00]	0.63 [0.24, 0.91]	0.76 [0.69, 0.82]
**Overall sensitivity, specificity, PPV, NPV (after outliers' exclusion)**							**0.13 [0.04, 0.26]**	**0.97 [0.92, 0.99]**	**0.60 [0.38, 0.8]**	**0.75 [0.63, 0.86]**

* Outlier

HSROC overall sensitivity [95%CI] = 0.13 [0.05, 0.27], HSROC overall specificity [95%CI] = 0.97 [0.92, 0.98] after exclusion of outliers

Table S6a. The results of odds rations (OR) of each study in increasing the risk of nodule malignancy for lack of halo or presence of thick halo with 95% confidence intervals and forest plots.

Author	Year	n	OR [95%CI]	Weight
Seo HS et al.*	2009	126	0.70 [0.32, 1.55]	12.05%
Sillery JC et al.	2010	102	2.42 [1.09, 5.38]	12.05%
Lee EK et al.	2012	110	4.26 [1.77, 10.24]	11.71%
Lai X et al.	2013	111	3.63 [1.57, 8.36]	11.89%
Lee SH et al.*	2013	66	0.19 [0.04, 0.83]	9.05%
Pompili G et al.	2013	102	18.22 [4.75, 69.9]	9.61%
Zhang JZ et al.*	2014	88	14.61 [5.03, 42.44]	10.87%
Jeong SH et al.	2016	178	2.38 [0.77, 7.39]	10.57%
Kuo TC et al.	2019	188	1.71 [0.8, 3.65]	12.21%
	**Overall OR**		**2.62 [1.24, 5.53]**	

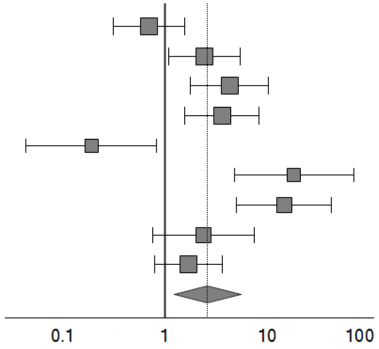

Test of heterogeneity
I^2=81.7%, p<0.0001

Egger's asymmetry test
b Egger=1.56, p=0.7007

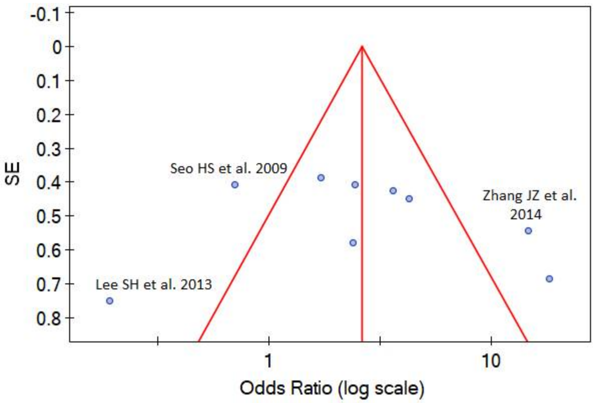

* Outlier

After exclusion of outliers: **overall OR [95%CI] = 3.34 [1.95, 5.73]**, test of heterogeneity I^2=51.2%, p=0.0685

Table S6b. The number of patients with true positive (TP), false negative (FN), false positive (FP), and true negative (TN) results presenting the estimates of sensitivity, specificity, positive predictive value (PPV), and negative predictive value (NPV with 95% confidence intervals of each study for lack of halo or presence of thick halo.

Author	Year	n	TP	FN	FP	TN	Sensitivity [95%CI]	Specificity [95%CI]	PPV [95%CI]	NPV [95%CI]
Seo HS et al. *	2009	126	46	20	46	14	0.70 [0.57, 0.80]	0.23 [0.13, 0.36]	0.50 [0.39, 0.61]	0.41 [0.25, 0.59]
Sillery JC et al.	2010	102	32	18	22	30	0.64 [0.49, 0.77]	0.58 [0.43, 0.71]	0.59 [0.45, 0.72]	0.63 [0.47, 0.76]
Lee EK et al.	2012	110	23	10	27	50	0.70 [0.51, 0.84]	0.65 [0.53, 0.75]	0.46 [0.32, 0.61]	0.83 [0.71, 0.92]
Lai X et al.	2013	111	25	12	27	47	0.68 [0.50, 0.82]	0.64 [0.52, 0.74]	0.48 [0.34, 0.62]	0.80 [0.67, 0.89]
Lee SH et al. *	2013	66	11	5	46	4	0.69 [0.41, 0.89]	0.08 [0.02, 0.19]	0.19 [0.10, 0.32]	0.44 [0.14, 0.79]
Pompili G et al.	2013	102	8	6	6	82	0.57 [0.29, 0.82]	0.93 [0.86, 0.97]	0.57 [0.29, 0.82]	0.93 [0.86, 0.97]
Zhang JZ et al.	2014	88	25	11	7	45	0.69 [0.52, 0.84]	0.87 [0.74, 0.94]	0.78 [0.60, 0.91]	0.80 [0.68, 0.90]
Jeong SH et al.	2016	178	18	4	102	54	0.82 [0.60, 0.95]	0.35 [0.27, 0.43]	0.15 [0.09, 0.23]	0.93 [0.83, 0.98]
Kuo TC et al.	2019	188	38	11	93	46	0.78 [0.63, 0.88]	0.33 [0.25, 0.42]	0.29 [0.21, 0.38]	0.81 [0.68, 0.90]
After outliers exclusion **overall sensitivity, specificity, PPV, NPV**							**0.70 [0.64, 0.76]**	**0.63 [0.43, 0.82]**	**0.46 [0.29, 0.63]**	**0.83 [0.75, 0.90]**

* Outlier

HSROC overall sensitivity and specificity are missing because of estimated variance components too close to the boundary of the parameter space

Table S7a. The results of odds rations (OR) of each study in increasing the risk of nodule malignancy when macrocalcifications, eggshell or rim calcifications are present in the ultrasound with 95% confidence intervals and forest plots.

Author	Year	n	OR [95%CI]	Weight
Seo HS et al.	2009	126	3.43 [1.05, 11.2]	15.86%
Lai X et al.	2013	111	1.00 [0.24, 4.25]	12.67%
Aydın Y et al.	2014	88	1.29 [0.14, 12.11]	6.86%
Yoon JH et al.	2014	177	2.22 [0.66, 7.53]	15.34%
Jeong SH et al.	2016	178	2.40 [0.79, 7.35]	16.77%
Kuo TC et al.	2019	188	14.89 [4.64, 47.75]	16.09%
Liu BJ et al.	2020	90	5.19 [1.65, 16.26]	16.40%
**Overall OR**			**3.28 [1.69, 6.35]**	

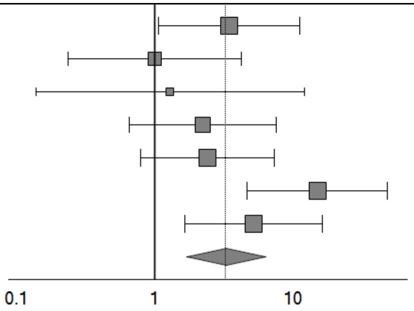

Test of heterogeneity
$I^2 = 45.4\%$, $p = 0.0885$

Egger's asymmetry test
b Egger = -3.09, p = 0.2966

Table S7b. The number of patients with true positive (TP), false negative (FN), false positive (FP), and true negative (TN) results presenting the estimates of sensitivity, specificity, positive predictive value (PPV), and negative predictive value (NPV with 95% confidence intervals of each study for macrocalcifications, eggshell or rim calcifications in the ultrasound.

Author	Year	n	TP	FN	FP	TN	Sensitivity [95%CI]	Specificity [95%CI]	PPV [95%CI]	NPV [95%CI]
Seo HS et al.	2009	126	13	53	4	56	0.20 [0.11, 0.31]	0.93 [0.84, 0.98]	0.76 [0.5, 0.93]	0.51 [0.42, 0.61]
Lai X et al.	2013	111	3	34	6	68	0.08 [0.02, 0.22]	0.92 [0.83, 0.97]	0.33 [0.07, 0.7]	0.67 [0.57, 0.76]
Aydın Y et al.	2014	88	1	5	11	71	0.17 [0.00, 0.64]	0.87 [0.77, 0.93]	0.08 [0.00, 0.38]	0.93 [0.85, 0.98]
Yoon JH et al.	2014	177	4	21	12	140	0.16 [0.05, 0.36]	0.92 [0.87, 0.96]	0.25 [0.07, 0.52]	0.87 [0.81, 0.92]
Jeong SH et al.	2016	178	5	17	17	139	0.23 [0.08, 0.45]	0.89 [0.83, 0.94]	0.23 [0.08, 0.45]	0.89 [0.83, 0.94]
Kuo TC et al.	2019	188	15	34	4	135	0.31 [0.18, 0.45]	0.97 [0.93, 0.99]	0.79 [0.54, 0.94]	0.8 [0.73, 0.86]
Liu BJ et al.	2020	90	10	18	6	56	0.36 [0.19, 0.56]	0.9 [0.8, 0.96]	0.63 [0.35, 0.85]	0.76 [0.64, 0.85]
**Overall sensitivity, specificity, PPV, NPV**							**0.21 [0.14, 0.29]**	**0.92 [0.89, 0.95]**	**0.44 [0.22, 0.67]**	**0.79 [0.68, 0.88]**

HSROC overall sensitivity [95%CI] = 0.2 [0.14, 0.28], HSROC overall specificity[95%CI] = 0.92 [0.89, 0.94]

Table S8a. The results of odds rations (OR) of each study in increasing the risk of nodule malignancy when any type of calcifications is present in the ultrasound with 95% confidence intervals and forest plots.

Author	Year	n	OR [95%CI]	Weight
Seo HS et al.	2009	126	5.14 [1.93, 13.72]	8.63%
Sillery JC et al.	2010	102	2.66 [0.65, 10.93]	5.94%
Lee EK et al.	2012	110	5.00 [1.96, 12.75]	8.97%
Lai X et al.	2013	111	4.36 [1.71, 11.12]	8.98%
Lee KH et al.	2013	75	0.67 [0.13, 3.41]	4.94%
Lee SH et al.	2013	66	22.27 [2.36, 210.14]	3.10%
Aydın Y et al.	2014	88	0.51 [0.06, 4.63]	3.20%
Cordes M et al.	2014	57	4.12 [0.94, 18.05]	5.63%
Yoon JH et al.	2014	177	4.16 [1.47, 11.79]	8.19%
Zhang JZ et al.*	2014	88	15.00 [4.46, 50.48]	7.06%
Cordes M et al..	2016	200	2.07 [1.18, 3.64]	12.03%
Jeong SH et al.	2016	178	2.00 [0.66, 6.02]	7.78%
Kuo TC et al.*	2019	188	12.99 [4.44, 38.03]	7.97%
Liu BJ et al.	2020	90	6.04 [1.94, 18.75]	7.57%
	**Overall OR**		**4.06 [2.6, 6.33]**	

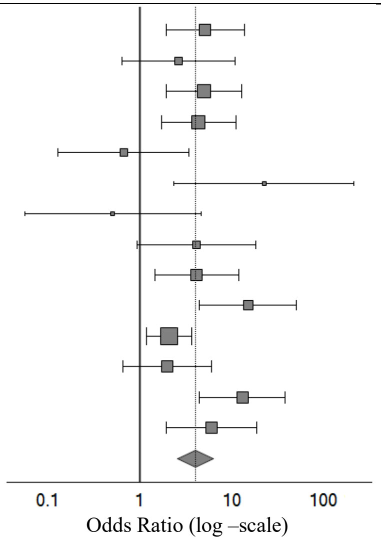

Test of heterogeneity
$I^2 = 52.4\%$, p = 0.0112

Egger's asymmetry test
b Egger = 0.66, p = 0.5855

*Outlier
After exclusion of outliers: **overall OR [95%CI] = 3.26 [2.2, 4.83],** test of heterogeneity I^2=30.6%, p=0.1469

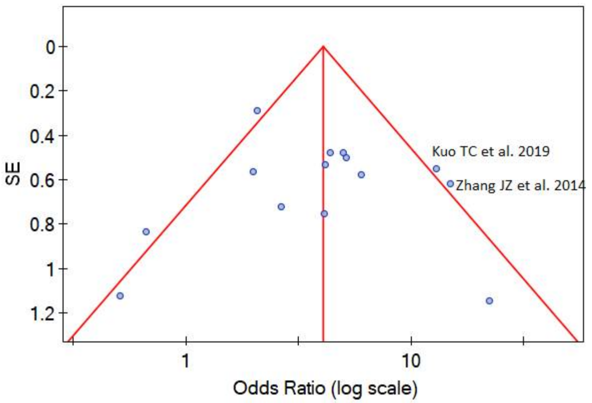

Table S8b. The number of patients with true positive (TP), false negative (FN), false positive (FP), and true negative (TN) results presenting the estimates of sensitivity, specificity, positive predictive value (PPV), and negative predictive value (NPV with 95% confidence intervals of each study for all types of calcifications in the ultrasound.

Author	Year	n	TP	FN	FP	TN	Sensitivity [95%CI]	Specificity [95%CI]	PPV [95%CI]	NPV [95%CI]
Seo HS et al.	2009	126	24	42	6	54	0.36 [0.25, 0.49]	0.90 [0.79, 0.96]	0.80 [0.61, 0.92]	0.56 [0.46, 0.66]
Sillery JC et al.	2010	102	7	43	3	49	0.14 [0.06, 0.27]	0.94 [0.84, 0.99]	0.70 [0.35, 0.93]	0.53 [0.43, 0.64]
Lee EK et al.	2012	110	15	18	11	66	0.45 [0.28, 0.64]	0.86 [0.76, 0.93]	0.58 [0.37, 0.77]	0.79 [0.68, 0.87]
Lai X et al.	2013	111	15	22	10	64	0.41 [0.25, 0.58]	0.86 [0.77, 0.93]	0.60 [0.39, 0.79]	0.74 [0.64, 0.83]
Lee KH et al.	2013	75	2	9	16	48	0.18 [0.02, 0.52]	0.75 [0.63, 0.85]	0.11 [0.01, 0.35]	0.84 [0.72, 0.93]
Lee SH et al.	2013	66	5	11	1	49	0.31 [0.11, 0.59]	0.98 [0.89, 1.00]	0.83 [0.36, 1.00]	0.82 [0.70, 0.90]
Aydın Y et al.	2014	88	1	5	23	59	0.17 [0.00, 0.64]	0.72 [0.61, 0.81]	0.04 [0.00, 0.21]	0.92 [0.83, 0.97]
Cordes M et al.	2014	57	7	17	3	30	0.29 [0.13, 0.51]	0.91 [0.76, 0.98]	0.70 [0.35, 0.93]	0.64 [0.49, 0.77]
Yoon JH et al.	2014	177	7	18	13	139	0.28 [0.12, 0.49]	0.91 [0.86, 0.95]	0.35 [0.15, 0.59]	0.89 [0.82, 0.93]
Zhang JZ et al.	2014	88	20	16	4	48	0.56 [0.38, 0.72]	0.92 [0.81, 0.98]	0.83 [0.63, 0.95]	0.75 [0.63, 0.85]
Cordes M et al.	2016	200	57	43	39	61	0.57 [0.47, 0.67]	0.61 [0.51, 0.71]	0.59 [0.49, 0.69]	0.59 [0.49, 0.68]
Jeong SH et al.	2016	178	5	17	20	136	0.23 [0.08, 0.45]	0.87 [0.81, 0.92]	0.2 [0.07, 0.41]	0.89 [0.83, 0.93]
Kuo TC et al.	2019	188	16	33	5	134	0.33 [0.20, 0.48]	0.96 [0.92, 0.99]	0.76 [0.53, 0.92]	0.80 [0.73, 0.86]
Liu BJ et al.	2020	90	11	17	6	56	0.39 [0.22, 0.59]	0.90 [0.80, 0.96]	0.65 [0.38, 0.86]	0.77 [0.65, 0.86]
**Overall sensitivity, specificity, PPV, NPV (after outliers' exclusion)**							**0.35 [0.27, 0.43]**	**0.88 [0.82, 0.92]**	**0.54 [0.39, 0.69]**	**0.76 [0.69, 0.83]**

HSROC overall sensitivity [95%CI] = 0.36 [0.29, 0.43], HSROC overall specificity [95%CI] = 0.89 [0.83, 0.92]

Table S9a. The results of odds rations (OR) of each study in increasing the risk of nodule malignancy for solitary nodule with 95% confidence intervals and forest plots.

Author	Year	n	OR [95%CI]	Weight
Pompili G et al.	2013	102	6 [1.27, 28.39]	24.3%
Cordes M et al.	2014	57	4.11 [0.45, 37.69]	12.0%
Zhang JZ et al.	2014	88	1.86 [0.71, 4.87]	63.7%
**Overall OR**			**2.72 [1.26, 5.86]**	

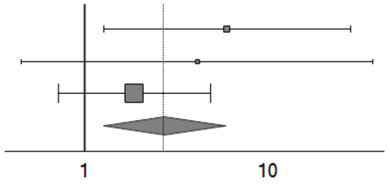

Odds Ratio (log –scale)

Test of heterogeneity
I^2=0.0%. p=0,4225

Egger's asymmetry test
b Egger=1.84, p=0.4194

Table S9b. The number of patients with true positive (TP), false negative (FN), false positive (FP), and true negative (TN) results presenting the estimates of sensitivity, specificity, positive predictive value (PPV), and negative predictive value (NPV with 95% confidence intervals of each study for solitary nodule.

Author	Year	n	TP	FN	FP	TN	Sensitivity [95%CI]	Specificity [95%CI]	PPV [95%CI]	NPV [95%CI]
Pompili G et al.	2013	102	12	2	44	44	0.86 [0.57, 0.98]	0.50 [0.39, 0.61]	0.21 [0.12, 0.34]	0.96 [0.85, 0.99]
Cordes M et al.	2014	57	23	1	28	5	0.96 [0.79, 1.00]	0.15 [0.05, 0.32]	0.45 [0.31, 0.60]	0.83 [0.36, 1.00]
Zhang JZ et al.	2014	88	12	24	11	41	0.33 [0.19, 0.51]	0.79 [0.65, 0.89]	0.52 [0.31, 0.73]	0.63 [0.50, 0.75]
**Overall sensitivity, specificity, PPV, NPV**							**0.74 [0.27, 1.00]**	**0.48 [0.17, 0.80]**	**0.38 [0.20, 0.58]**	**0.83 [0.51, 1.00]**

HSROC overall sensitivity and specificity are missing because HSROC requires a minimum of four studies

Table S10a. The results of odds rations (OR) of each study in increasing the risk of nodule malignancy for taller than wide feature in the ultrasound with 95% confidence intervals and forest plots.

Author	Year	n	OR [95%CI]	Weight
Lee KH et al.	2013	75	1.09 [0.05, 24.15]	8.40%
Cordes M et al.	2014	57	4.28 [0.17, 109.61]	7.68%
Yoon JH et al.	2014	177	4.32 [0.68, 27.26]	23.81%
Cordes M et al.	2016	200	1.00 [0.02, 50.89]	5.23%
Kuo TC et al.	2019	188	1.14 [0.21, 6.08]	28.86%
Liu BJ et al.	2020	90	5.00 [0.86, 29.12]	26.02%
	**Overall OR**		**2.52 [1.02, 6.19]**	

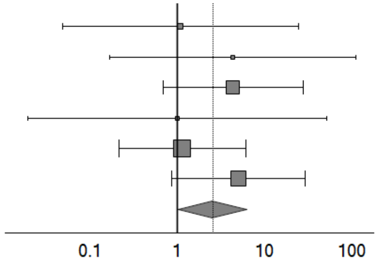

Odds Ratio (log –scale)

Test of heterogeneity
I^2=0.0%, p=0.7961

Egger's asymmetry test
b Egger=-0.46, p=0.6656

Table S10b. The number of patients with true positive (TP), false negative (FN), false positive (FP), and true negative (TN) results presenting the estimates of sensitivity, specificity, positive predictive value (PPV), and negative predictive value (NPV with 95% confidence intervals of each study for taller than wide feature in the ultrasound

Author	Year	n	TP	FN	FP	TN	Sensitivity [95%CI]	Specificity [95%CI]	PPV [95%CI]	NPV [95%CI]
Lee KH et al.	2013	75	0	11	2	62	0.00 [0.00, 0.28]	0.97 [0.89, 1.00]	0.00 [0.00, 0.97]	0.85 [0.75, 0.92]
Cordes M et al.	2014	57	1	23	0	33	0.04 [0.00, 0.21]	1.00 [0.89, 1.00]	1.00 [0.02, 1.00]	0.59 [0.45, 0.72]
Yoon JH et al.	2014	177	2	23	3	149	0.08 [0.01, 0.26]	0.98 [0.94, 1.00]	0.40 [0.05, 0.85]	0.87 [0.81, 0.91]
Cordes M et al.	2016	200	0	100	0	100	0.00 [0.00, 0.04]	1.00 [0.96, 1.00]	NA	0.50 [0.43, 0.57]
Kuo TC et al.	2019	188	2	47	5	134	0.04 [0.00, 0.14]	0.96 [0.92, 0.99]	0.29 [0.04, 0.71]	0.74 [0.67, 0.80]
Liu BJ et al.	2020	90	4	24	2	60	0.14 [0.04, 0.33]	0.97 [0.89, 1.00]	0.67 [0.22, 0.96]	0.71 [0.61, 0.81]
**Overall sensitivity, specificity, PPV, NPV**							**0.03 [0.00, 0.10]**	**0.98 [0.97, 1.00]**	**0.41 [0.14, 0.70]**	**0.72 [0.58, 0.84]**

NA – not applicable
HSROC overall sensitivity and specificity are missing because one or more studies have TP+FP=0 or TN+FN=0.

Table S11a. The results of odds rations (OR) of each study in increasing the risk of nodule malignancy for solid or mainly solid structure with 95% confidence intervals and forest plots.

Author	Year	n	OR [95%CI]	Weight
Seo HS et al.*	2009	126	106.4 [27.81, 407.11]	9.56%
Sillery JC et al.	2010	102	16.65 [0.92, 299.74]	5.55%
Lee EK et al.	2012	110	2.22 [0.10, 47.48]	5.21%
Lai X et al.	2013	111	2.42 [0.50, 11.84]	8.86%
Lee KH et al.	2013	75	1.50 [0.29, 7.68]	8.73%
Pompili G et al.	2013	102	1.86 [0.22, 15.62]	7.35%
Yoon JH et al.	2014	177	0.31 [0.05, 1.79]	8.38%
Zhang JZ et al.*	2014	88	115.73 [6.73, 1990.67]	5.65%
Jeong SH et al.	2016	178	8.20 [2.33, 28.84]	9.79%
Kuru B et al.	2018	139	3.55 [1.49, 8.45]	10.80%
Kuo TC et al.	2019	188	1.13 [0.43, 3.03]	10.53%
Liu BJ et al.	2020	90	2.66 [0.7, 10.07]	9.59%
**Overall OR**			**4.15 [1.63, 10.55]**	

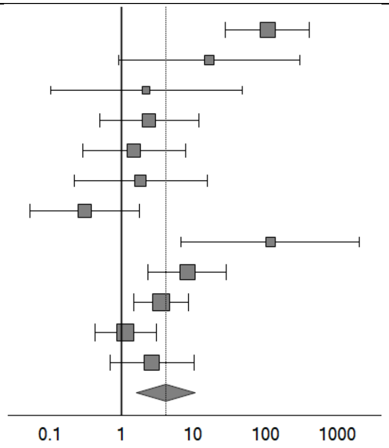

Test of heterogeneity
$I^2 = 76.9\%$, $p<0.0001$

Egger's asymmetry test
b Egger = 0.83, p = 0.6422

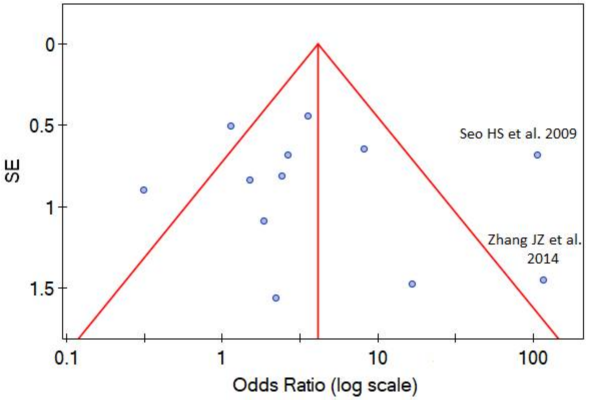

* Outlier

After exclusion of outliers: **overall OR [95%CI] = 2.3 [1.27, 4.17]**, test of heterogeneity $I^2 = 35.8\%$, p = 0.1217

Table S11b. The number of patients with true positive (TP), false negative (FN), false positive (FP), and true negative (TN) results presenting the estimates of sensitivity, specificity, positive predictive value (PPV), and negative predictive value (NPV with 95% confidence intervals of each study for solid or mainly solid structure.

Author	Year	n	TP	FN	FP	TN	Sensitivity [95%CI]	Specificity [95%CI]	PPV [95%CI]	NPV [95%CI]
Seo HS et al. *	2009	126	56	10	3	57	0.85 [0.74, 0.92]	0.95 [0.86, 0.99]	0.95 [0.86, 0.99]	0.85 [0.74, 0.93]
Sillery JC et al.	2010	102	50	0	45	7	1.00 [0.93, 1.00]	0.13 [0.06, 0.26]	0.53 [0.42, 0.63]	1.00 [0.59, 1.00]
Lee EK et al.	2012	110	33	0	75	2	1.00 [0.89, 1.00]	0.03 [0.00, 0.09]	0.31 [0.22, 0.40]	1.00 [0.03, 1.00]
Lai X et al.	2013	111	35	2	65	9	0.95 [0.82, 0.99]	0.12 [0.06, 0.22]	0.35 [0.26, 0.45]	0.82 [0.48, 0.98]
Lee KH et al.	2013	75	9	2	48	16	0.82 [0.48, 0.98]	0.25 [0.15, 0.37]	0.16 [0.07, 0.28]	0.89 [0.65, 0.99]
Pompili G et al.	2013	102	13	1	77	11	0.93 [0.66, 1.00]	0.13 [0.06, 0.21]	0.14 [0.08, 0.23]	0.92 [0.62, 1.00]
Yoon JH et al.	2014	177	23	2	148	4	0.92 [0.74, 0.99]	0.03 [0.01, 0.07]	0.13 [0.09, 0.19]	0.67 [0.22, 0.96]
Zhang JZ et al. *	2014	88	36	0	20	32	1.00 [0.90, 1.00]	0.62 [0.47, 0.75]	0.64 [0.50, 0.77]	1.00 [0.89, 1.00]
Jeong SH et al.	2016	178	19	3	68	88	0.86 [0.65, 0.97]	0.56 [0.48, 0.64]	0.22 [0.14, 0.32]	0.97 [0.91, 0.99]
Kuru B et al.	2018	139	43	8	53	35	0.84 [0.71, 0.93]	0.40 [0.29, 0.51]	0.45 [0.35, 0.55]	0.81 [0.67, 0.92]
Kuo TC et al.	2019	188	43	6	120	19	0.88 [0.75, 0.95]	0.14 [0.08, 0.21]	0.26 [0.20, 0.34]	0.76 [0.55, 0.91]
Liu BJ et al.	2020	90	25	3	47	15	0.89 [0.72, 0.98]	0.24 [0.14, 0.37]	0.35 [0.24, 0.47]	0.83 [0.59, 0.96]
**Overall sensitivity, specificity, PPV, NPV (after outliers' exclusion)**							**0.93 [0.87, 0.97]**	**0.18 [0.08, 0.31]**	**0.28 [0.20, 0.37]**	**0.9 [0.81, 0.96]**

* Outlier

HSROC overall sensitivity [95%CI] = 0.92 [0.87, 0.96], HSROC overall specificity[95%CI] = 0.15 [0.08, 0.27] after exclusion of outliers

Table S12a. The results of odds rations (OR) of each study in increasing the risk of nodule malignancy for size over 4 cm with 95% confidence intervals and forest plots.

Author	Year	n	OR [95%CI]	Weight
Seo HS et al.	2009	126	1.60 [0.74, 3.48]	22.1%
Kamran SC et al.	2013	7348	1.25 [1.02, 1.55]	37.0%
Zhang JZ et al.	2014	88	1.37 [0.47, 3.96]	16.0%
Kim M et al.	2018	160	1.28 [0.36, 4.59]	12.7%
Kuru B et al.	2018	139	9.69 [2.61, 36.01]	12.2%
**Overall OR**			**1.73 [0.99, 3.00]**	

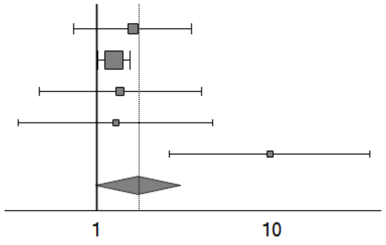

Odds Ratio (log –scale)

Test of heterogeneity
I^2=57.09%, p=0.0536

Egger's asymmetry test
b Egger=1.25, p=0.2732

Table S12b. The number of patients with true positive (TP), false negative (FN), false positive (FP), and true negative (TN) results presenting the estimates of sensitivity, specificity, positive predictive value (PPV), and negative predictive value (NPV with 95% confidence intervals of each study for size over 4 cm.

Author	Year	n	TP	FN	FP	TN	Sensitivity [95%CI]	Specificity [95%CI]	PPV [95%CI]	NPV [95%CI]
Seo HS et al.	2009	126	23	43	15	45	0.35 [0.24, 0.48]	0.75 [0.62, 0.85]	0.61 [0.43, 0.76]	0.51 [0.40, 0.62]
Kamran SC et al.	2013	7348	116	811	657	5764	0.13 [0.10, 0.15]	0.90 [0.89, 0.90]	0.15 [0.13, 0.18]	0.88 [0.87, 0.88]
Zhang JZ et al.	2014	88	8	28	9	43	0.22 [0.10, 0.39]	0.83 [0.70, 0.92]	0.47 [0.23, 0.72]	0.61 [0.48, 0.72]
Kim M et al.	2018	160	4	46	7	103	0.08 [0.02, 0.19]	0.94 [0.87, 0.97]	0.36 [0.11, 0.69]	0.69 [0.61, 0.76]
Kuru B et al.	2018	139	13	38	3	85	0.25 [0.14, 0.40]	0.97 [0.90, 0.99]	0.81 [0.54, 0.96]	0.69 [0.60, 0.77]
**Overall sensitivity, specificity, PPV, NPV**							**0.19 [0.11, 0.30]**	**0.89 [0.83, 0.94]**	**0.47 [0.17, 0.77]**	**0.69 [0.51, 0.84]**

HSROC overall sensitivity[95%CI] =0.18 [0.11, 0.28], HSROC Overall specificity [95%CI] = 0.89 [0.81, 0.94] after exclusion of outliers

Table S13a. The results of odds rations (OR) of each study in increasing the risk of nodule malignancy for heterogeoenous echostructure in the ultrasound with 95% confidence intervals and forest plots.

Author	Year	n	OR [95%CI]	Weight
Sillery JC et al.	2010	102	1.6 [0.73, 3.54]	14.67%
Lai X et al.	2013	111	1.73 [0.78, 3.82]	14.65%
Lee SH et al.	2013	66	1.78 [0.57, 5.53]	13.01%
Cordes M et al.	2014	57	5.16 [1.28, 20.77]	11.72%
Zhang JZ et al.*	2014	88	21 [6.88, 64.06]	13.12%
Cordes M et al.*	2016	200	245.18 [14.82, 4056.92]	6.14%
Jeong SH et al.	2016	178	0.9 [0.36, 2.28]	14.03%
Kuo TC et al.	2019	188	0.87 [0.26, 2.92]	12.65%
**Overall OR**			**3.07 [1.28, 7.36]**	

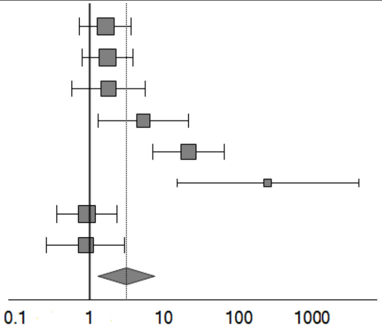

Test of heterogeneity
$I^2 = 79.9\%$. $p<0.0001$

Odds Ratio (log –scale)

Egger's asymmetry test
b Egger = 4.47, p = 0.0976

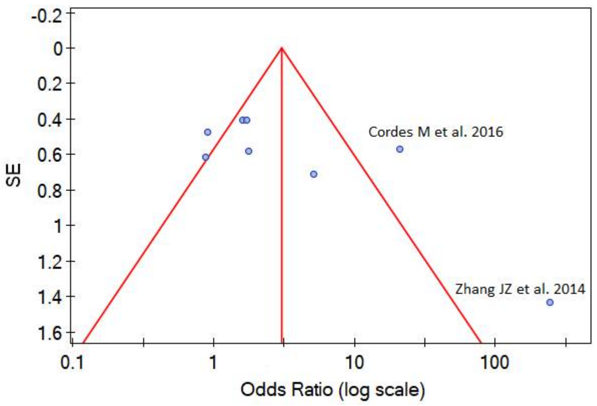

* Outlier
After exclusion of outliers: **overall OR [95%CI] = 1.53 [1.02, 2.3]**, test of heterogeneity $I^2 = 3.32\%$, p = 0.3953

Table S13b. The number of patients with true positive (TP), false negative (FN), false positive (FP), and true negative (TN) results presenting the estimates of sensitivity, specificity, positive predictive value (PPV), and negative predictive value (NPV with 95% confidence intervals of each study for heterogeoenous echostructure.

Author	Year	n	TP	FN	FP	TN	Sensitivity [95%CI]	Specificity [95%CI]	PPV [95%CI]	NPV [95%CI]
Sillery JC et al.	2010	102	24	26	19	33	0.48 [0.34, 0.63]	0.63 [0.49, 0.76]	0.56 [0.40, 0.71]	0.56 [0.42, 0.69]
Lai X et al.	2013	111	20	17	30	44	0.54 [0.37, 0.71]	0.59 [0.47, 0.71]	0.40 [0.26, 0.55]	0.72 [0.59, 0.83]
Lee SH et al.	2013	66	9	7	21	29	0.56 [0.30, 0.80]	0.58 [0.43, 0.72]	0.30 [0.15, 0.49]	0.81 [0.64, 0.92]
Cordes M et al. *	2014	57	21	3	19	14	0.88 [0.68, 0.97]	0.42 [0.25, 0.61]	0.53 [0.36, 0.68]	0.82 [0.57, 0.96]
Zhang JZ et al. *	2014	88	30	6	10	42	0.83 [0.67, 0.94]	0.81 [0.67, 0.90]	0.75 [0.59, 0.87]	0.88 [0.75, 0.95]
Cordes M et al.	2016	200	100	0	45	55	1.00 [0.96, 1.00]	0.55 [0.45, 0.65]	0.69 [0.61, 0.76]	1.00 [0.94, 1.00]
Jeong SH et al.	2016	178	14	8	103	53	0.64 [0.41, 0.83]	0.34 [0.27, 0.42]	0.12 [0.07, 0.19]	0.87 [0.76, 0.94]
Kuo TC et al.*	2019	188	45	4	129	10	0.92 [0.80, 0.98]	0.07 [0.04, 0.13]	0.26 [0.20, 0.33]	0.71 [0.42, 0.92]
**Overall sensitivity, specificity, PPV, NPV (after outliers' exclusion)**							**0.69 [0.33, 0.96]**	**0.53 [0.41, 0.65]**	**0.4 [0.16, 0.67]**	**0.82 [0.63, 0.96]**

*Outlier
HSROC overall sensitivity [95%CI] = 0.77 [0.34, 0.96], HSROC overall specificity [95%CI] = 0.53 [0.43, 0.63] after exclusion of outliers

Table S14a. The results of odds rations (OR) of each study in increasing the risk of nodule malignancy for Doppler pattern 3 or more with 95% confidence intervals and forest plots.

Author	Year	n	OR [95%CI]	Weight
Sillery JC et al.	2010	100	1.8 [0.56, 5.81]	16.6%
Lai X et al.	2013	90	2.9 [0.77, 11.01]	14.3%
Lee SH et al.	2013	66	0.92 [0.25, 3.46]	14.4%
Pompili G et al.	2013	102	4.72 [0.71, 31.21]	8.8%
Cordes M et al..	2016	200	0.73 [0.4, 1.32]	28.0%
Jeong SH et al.	2016	175	6.23 [0.81, 47.96]	7.8%
Yang GCH et al.	2016	279	0.55 [0.1, 3.08]	10.1%
	**Overall OR**		**1.44 [0.76, 2.74]**	

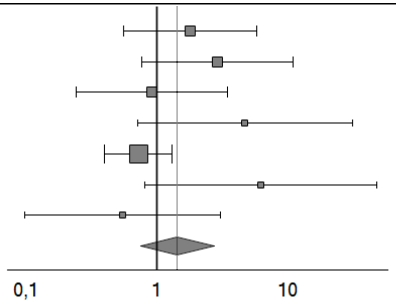

Test of heterogeneity
$I^2 = 42.8\%$, p = 0.1125

Egger's asymmetry test
b Egger = 2.03, p = 0.0725

Table S15b. The number of patients with true positive (TP), false negative (FN), false positive (FP), and true negative (TN) results presenting the estimates of sensitivity, specificity, positive predictive value (PPV), and negative predictive value (NPV with 95% confidence intervals of each study for Doppler pattern 3 or more.

Author	Year	n	TP	FN	FP	TN	Sensitivity [95%CI]	Specificity [95%CI]	PPV [95%CI]	NPV [95%CI]
Sillery JC et al.	2010	100	43	5	43	9	0.90 [0.77, 0.97]	0.17 [0.08, 0.30]	0.50 [0.39, 0.61]	0.64 [0.35, 0.87]
Lai X et al.	2013	90	28	3	45	14	0.90 [0.74, 0.98]	0.24 [0.14, 0.37]	0.38 [0.27, 0.50]	0.82 [0.57, 0.96]
Lee SH et al.	2013	66	4	19	8	35	0.17 [0.05, 0.39]	0.81 [0.67, 0.92]	0.33 [0.10, 0.65]	0.65 [0.51, 0.77]
Pompili G et al.	2013	102	2	12	3	85	0.14 [0.02, 0.43]	0.97 [0.90, 0.99]	0.40 [0.05, 0.85]	0.88 [0.79, 0.93]
Cordes M et al.	2016	200	29	71	36	64	0.29 [0.20, 0.39]	0.64 [0.54, 0.73]	0.45 [0.32, 0.57]	0.47 [0.39, 0.56]
Jeong SH et al.	2016	175	21	1	118	35	0.95 [0.77, 1.00]	0.23 [0.16, 0.30]	0.15 [0.10, 0.22]	0.97 [0.85, 1.00]
Yang GCH et al.	2016	279	4	2	214	59	0.67 [0.22, 0.96]	0.22 [0.17, 0.27]	0.02 [0.01, 0.05]	0.97 [0.89, 1.00]
**Overall sensitivity, specificity, PPV, NPV**							**0.60 [0.29, 0.88]**	**0.48 [0.23, 0.74]**	**0.28 [0.10, 0.51]**	**0.80 [0.61, 0.94]**

HSROC overall sensitivity[95%CI] = 0.59 [0.24, 0.87], HSROC overall specificity[95%CI] = 0.49 [0.22, 0.76]
